# A Blade Tip Timing Method Based on a Microwave Sensor

**DOI:** 10.3390/s17051097

**Published:** 2017-05-11

**Authors:** Jilong Zhang, Fajie Duan, Guangyue Niu, Jiajia Jiang, Jie Li

**Affiliations:** 1 State Key Laboratory of Precision Measuring Technology and Instruments; Tianjin University, Tianjin 300072, China; zhangjilong@tju.edu.cn (J.Z.); niuguangyue@tju.edu.cn (G.N.); jiajiajiang@tju.edu.cn (J.J.); 2 China Gas Turbine Establishment; Chengdu 610500, China; lijie08160816@163.com

**Keywords:** blade tip timing, blade vibration measurement, microwave sensor

## Abstract

Blade tip timing is an effective method for blade vibration measurements in turbomachinery. This method is increasing in popularity because it is non-intrusive and has several advantages over the conventional strain gauge method. Different kinds of sensors have been developed for blade tip timing, including optical, eddy current and capacitance sensors. However, these sensors are unsuitable in environments with contaminants or high temperatures. Microwave sensors offer a promising potential solution to overcome these limitations. In this article, a microwave sensor-based blade tip timing measurement system is proposed. A patch antenna probe is used to transmit and receive the microwave signals. The signal model and process method is analyzed. Zero intermediate frequency structure is employed to maintain timing accuracy and dynamic performance, and the received signal can also be used to measure tip clearance. The timing method uses the rising and falling edges of the signal and an auto-gain control circuit to reduce the effect of tip clearance change. To validate the accuracy of the system, it is compared experimentally with a fiber optic tip timing system. The results show that the microwave tip timing system achieves good accuracy.

## 1. Introduction

Blades are the key components of large rotating machines such as aero engines and gas turbines. Measuring the vibration parameters of blades under working conditions is crucial for analyzing dynamic behavior and damage and stress failure mechanisms, as well as for estimating blade lifespan [1,2]. The blade tip timing (BTT) method has been widely used in blade vibration measurement and analysis instead of the conventional strain gauge system, because it is non-intrusive and is able to measure the vibrations of all blades in real time [3,4].

**Figure 1:**
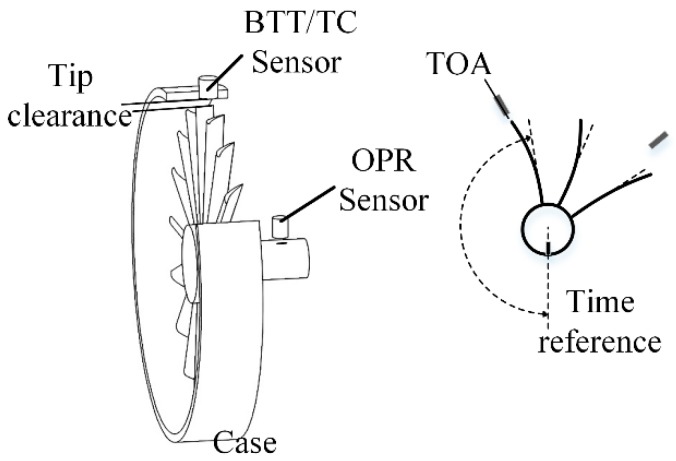
Blade tip timing method.

The BTT system measures the time of arrival (TOA) of a blade tip at the BTT sensor. The TOA changes because of blade vibrations. A once-per-revolution sensor is mounted near the shaft to generate a reference timing signal, as shown in Figure 1. The TOA data is converted to the vibration displacement, and parameters such as vibration frequency and mode can be analyzed through different identification algorithms [5,6,7]. Tip clearance—the gap between blade tip and engine casing—is an important parameter to guarantee the efficiency and safety of the turbine engines [8].

The TOA can be acquired by different kinds of sensors such as optical sensors, capacitive sensors, eddy current sensors and magnetoresistive sensors. Optical sensors have achieved good time accuracy and spatial resolution, with small size and enough bandwidth, using the amplitude of the reflected signal or laser Doppler technology [9,10,11,12,13]. Good time accuracy has also been observed in eddy current sensors [14,15,16] and magnetoresistive sensors [17]. However, high operating temperatures, as well as smoke particles and debris in the gas medium make optical sensors unsuitable for many aero engines and gas turbines. Capacitance sensors can be used in high temperature environments; however, they are sensitive to the variation of gas permittivity, and their bandwidth is limited [18,19]. Microwave sensors are a promising technology to overcome these limitations. Microwave sensors are not sensitive to a polluted medium and have a sufficiently large bandwidth. Microwave tip clearance sensors have been reported to work in conditions up to 2000 °C [20].

Different kinds of microwave sensors have been developed for blade tip clearance and blade tip timing measurements [20,21,22,23,24]. The waveguide systems use a metal waveguide as an antenna, and the operating temperature can be very high [21,22]. However, it needs an additional reference channel for the tip clearance measurement, and a higher frequency or dielectric filling is needed to control the diameter of the sensor. The coaxial resonator sensor achieves good spatial resolution for tip timing, and is unsuited for tip clearance measurement [24]. The patch antenna can achieve tip timing and tip clearance measurement simultaneously without an additional reference channel.

In the present article, a microwave system for BTT measurement is introduced. A microwave sensor based on a patch antenna is used to send and receive microwave signals. The sensor can realize BTT and tip clearance measurement simultaneously. The form of the received signal and the processing method is analyzed. A timing method for the microwave sensor is proposed to reduce the effect of tip clearance change on the BTT measurement. Experiments are conducted to compare the microwave BTT system with an existing fiber optic system to verify the accuracy of the microwave system. The system is designed to realize simultaneous BTT and tip clearance measurements in the same system; the present paper is focused on the BTT measurement aspect.

## 2. Microwave BTT System

### 2.1. System Overview and Signal Analysis

**Figure 2:**
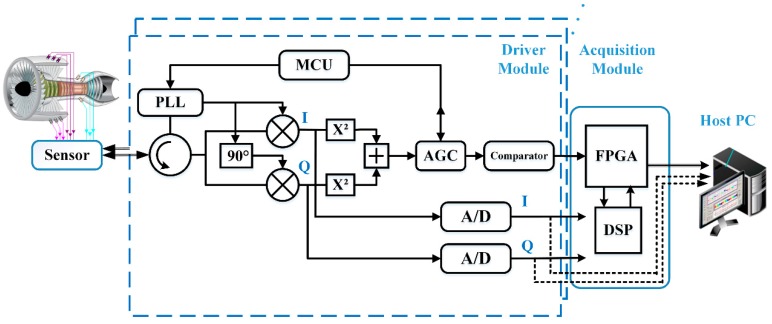
Structure of the microwave blade tip timing and tip clearance system.

The BTT measurement system acquires the TOA of engine blades using microwave sensors. The system structure is shown in Figure 2, with the amplifiers and filters omitted. The phase-locked frequency source outputs a precise microwave signal, the signal is amplified and transmitted to the probe through the circulator, and thus generates the orthogonal local oscillator signals. The probe projects the microwave signal to the blade tip and receives the reflected signal. The received signal is filtered and quadrature down-converted by the mixers and a pair of intermediate frequency (IF) signals is generated. The sum of their squares is calculated in circuits to generate an amplitude signal, and the two IF signals are also sampled by analog-to-digital converter (ADC) for tip clearance measurement. The amplitude signal is amplified through the automatic gain control (AGC) circuit and the timing pulse is generated by the comparator. The timing pulse signals of different channels are acquired and the TOAs of all the blades are calculated and uploaded to the host by the acquisition module.

Assuming the transmitting signal generated by the source is *X*_0_ *= A_s_*cos(*ω_s_t + φ*_0_), the received signal is:

(1) $$Y=A(t)\cos(w_{s}t+\varphi_{0}+\varphi_{l}+\varphi (t))+A_{r}\cos(w_{s}t+\varphi_{l}),$$

where *φ_l_* is the accumulated phase in the transmission path and *φ*(*t*) is the phase difference caused by the change of tip clearance. *A_r_*cos(*ω_s_t + φ_l_*) expresses the reflected signal from the probe itself, and its amplitude changes slowly with the drift of the antenna reflection coefficient. This signal should be removed in the signal processing. According to the Friis transmission equation, the sensor received power of the reflected microwave signal can be expressed as:

(2) $$P_{r}=\frac{P_{t}G_{t}A_{e}\sigma }{{\left(4\pi \right)}^{2}R^{4}},$$

where *P_t_* is the transmitting power, *G_t_* is the antenna gain of the sensor, *A_e_* is the effective area of the receiving antenna, *σ* is the radar cross-section (RCS) of the blade, and *R* is the distance between the sensor and the blade tip.

When the blade is perpendicular to the face of the sensor, assume the maximum amplitude of the received signal is *A*_0_ and the clearance between the sensor and blade tip is *d*_0_. So, according to Equation (2), the amplitude of the received signal can be expressed as:

(3) $$A(t)=\frac{d_{0}{}^{2}}{d^{2}(t)}\cdot \sqrt{\frac{\sigma (t)}{\sigma_{0}}}\cdot A_{0},$$

where *d*(*t*) and *σ*(*t*) are the clearance and the RCS, respectively, when the blade is in different positions. The received radio-frequency signal needs to be down-converted. If the heterodyne method is adopted, the form of the IF signal would be:

(4) $$Y_{IF}=A(t)\cos(w_{i}t+\varphi_{0}+\varphi_{l}+\varphi (t))+A_{r}\cos(w_{i}t+\varphi_{l}).$$

The reflected signal from the probe itself cannot be eliminated by the filter. Also, the phase factor is retained, so the amplitude and the shape of the signal are affected by phase variation. Thus, the IF *w_i_* must be adjusted in real time to maintain the proper signal waveform [23]. The change of IF also affects BTT accuracy, so the zero-IF quadrature demodulation method is employed in the signal processing. The received signal is mixed with the local oscillator signal *A_i_*cos(*ω_s_t + φ_i_*) and the orthogonal local oscillator signal *A_i_*sin(*ω_s_t + φ_i_*), respectively, and subsequently passes the low-pass filter. The reflected signal from the probe itself *A_r_A_i_*cos(*φ_l_* − *φ_i_*) usually changes slowly, and is filtered together with the other direct current component of the circuit. The result is:

(5) $$S_{I}=\frac{d_{0}{}^{2}}{d^{2}(t)}\cdot \sqrt{\frac{\sigma (t)}{\sigma_{0}}}\cdot A_{0}A_{i}\cos(\varphi_{0}+\varphi_{l}+\varphi (t)-\varphi_{i}),$$

and the orthogonal signal *S_Q_.* Squaring *S_I_* and *S_Q_* and adding them together gives:

(6) $$S(t)=\frac{d_{0}{}^{4}}{d^{4}(t)}\cdot \frac{\sigma (t)}{\sigma_{0}}\cdot A_{0}^{2}A_{i}^{2}.$$

The phase component is removed and the signal edge is sharper, so the signal after processing is suitable for BTT. Considering that the change in clearance is small when the blade tip passes the sensor, the main factor is the change of the blade tip RCS. The maximum amplitude of *S*(*t*) is also inversely proportional to *d*_0_. The received signal is modulated by the blade passing the probe. The system bandwidth is mainly determined by circuit devices, because the carrier frequency is high enough. The bandwidth of the proposed system is 10 MHz.

For a flat plate whose area is *A*, *σ* = 4*πA*^2^*/λ*^2^ when the beam is perpendicular to the plate. The angle of incidence has little change when the blade passes, so *σ*(*t*) mainly reflects the change of the projected area of the microwave beam on the blade tip. For a free blade with a flat tip, the projected area of the microwave beam on the blade tip can be used to approximate the RCS. A small sensor diameter and beam width mean a short rising time of the signal, or good spatial resolution, which is preferred for BTT.

The phase in Equation (5) can be obtained from *S_i_* and *S_q_* by arctangent or other algorithms. The phase drift *φ_l_* in the transmission path due to factors such as temperature can be obtained by tuning the frequency out of the sensor bandwidth. Thus, tip clearance can be acquired from *φ*(*t*) after calibration. This is the principle of tip clearance measurement based on a microwave sensor. Thus, it is feasible to integrate BTT and tip clearance measurements into one system. The tip timing pulse can help to pick out part of the blade tip in the phase signal. The two measurement functions share the driver circuit, acquisition card, and use the same kind of microwave sensor, as shown in the lower part of Figure 2.

### 2.2. Microwave Sensor for BTT

**Figure 3:**
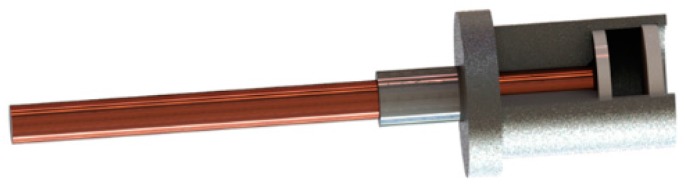
Structure of the sensor probe.

A 24-GHz microwave probe based on an end-fire patch antenna is used in the proposed BTT system. The circular patch antenna is mounted in a metal case with a ceramic cap in front of it, as shown in Figure 3. The outer diameter of the probe is 8 mm.

**Figure 4:**
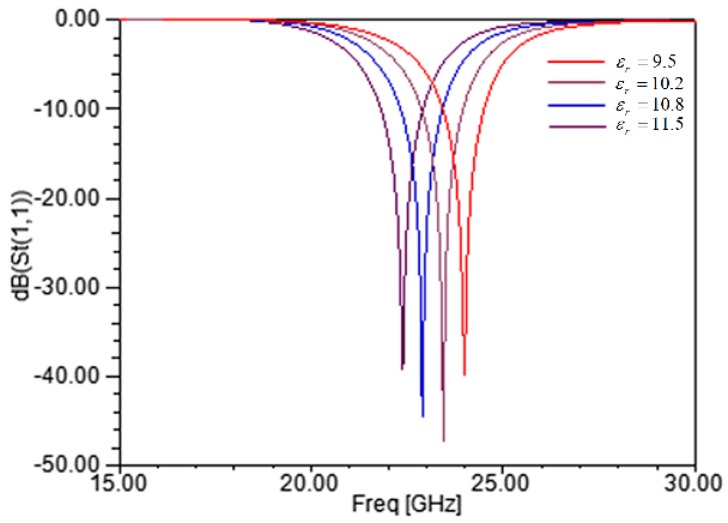
Reflection coefficient of the sensor with the permittivity increased by 20%.

The temperature in the turbine engine changes over a wide range during operation, and the probe pass band varies with temperature in a certain range. The main effect of temperature on the probe is the change in the relative permittivity of ceramic. Figure 4 shows the simulation result of the reflection coefficient when the permittivity of the ceramic substrate increases by 20%. The permittivity of the simulation curve rises by a 7% step. The result shows that the permittivity of the ceramic has a significance effect on the sensor characteristic. A recent probe prototype used alumina-based ceramic. The permittivity of pure alumina changes greatly with temperature, but the permittivity of alumina ceramic can remain nearly constant through adding materials with negative temperature coefficients of the dielectric constant. The output frequency of the signal source can be adjusted according to the amplitude of the received signal acquired by the AGC circuit; the variation is within the adjustable range of the driver circuit. In addition, the design of the sensor is also adjusted to increase the bandwidth appropriately and reduce the impact of permittivity. The prototype probe can operate at up to 600 °C. Silicon nitride has good thermal shock resistance; its thermal conductivity and coefficient of thermal expansion is lower than alumina, so it benefits the structural reliability. Furthermore, its temperature coefficient of the dielectric constant is lower. Thus, silicon nitride-based ceramic is a better choice for microwave probes used in high temperatures.

**Figure 5:**
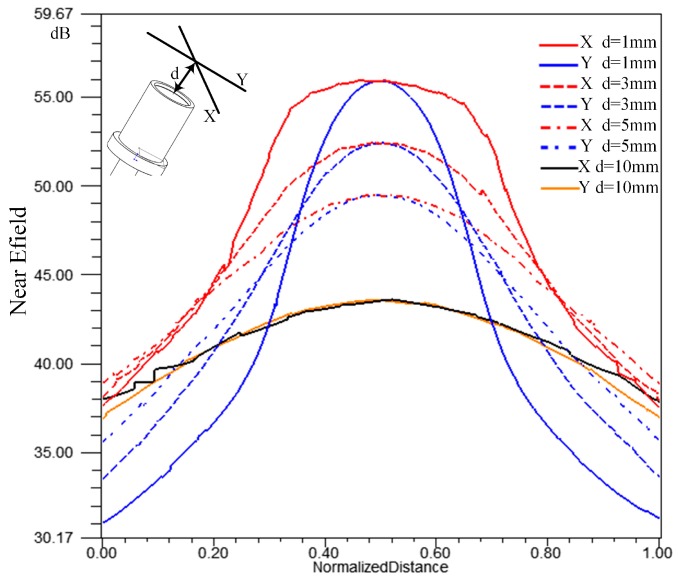
Electric field strengths along the two directions at different distances.

The near and far field of an antenna is usually distinguished by the Fraunhofer distance *d* = 2*D*^2^*/λ*, where *D* is the antenna diameter. The 24-GHz sensor works mainly in the near field. The radiation patterns of the near field change with distance from the antenna, and are more complex than those of the far field. The feed point of the patch antenna is usually not in the center of the antenna, in order to achieve impedance matching. Assume the direction of the line between the feed point and the center is X, and the direction Y is perpendicular to X and the axial direction of the probe. The simulation results of the electric field strengths along these two directions at different distances are shown in Figure 5. The length of the lines in the simulation are all 20 mm.

The results show that the field intensity distribution is not symmetrical in the near field due to the feed point offset. The beam is obviously wider in direction X than that in direction Y, and the differences decrease with the distance. The electric field strengths along the two directions are similar with each other when the distance is 10 mm, within the far field. So, the probe might be installed with the direction of the feed point consistent with the length direction of the blade tip, in order to reduce the signal rise time. The simulation shows that the large difference of the field distribution is mainly in the region close to the probe, within about 3 mm. Usually the probe is mounted in a small recess in the casing for safety, so a recess of 2 to 3 mm can be used to avoid the region and reduce the effect. Considering that the change of the blade twist angle is usually small, the effect can be minimized. The BTT sensor based on the patch antenna is designed to project microwave signals to the blade tip steadily, so it is also a tip clearance sensor, which is a reason to choose this structure. The opposite way is to make a strong coupling between the sensor and the blade, thereby the passband differs greatly, depending on whether the blade is in front of the sensor or not. The signal reflection due to impedance mismatch shows the arrival of the blades [24].

### 2.3. Timing Method

The TOA of every blade needs to be extracted from the blade tip signal. There are two main methods to generate the BTT pulse. One method involves comparing the rising or falling edge of the signal with a fixed trigger voltage in order to generate a pulse sequence for timing. The other involves finding the time of signal peaks; the zero-crossing point of the resulting differential signal is the peak of the original signal.

**Figure 6:**
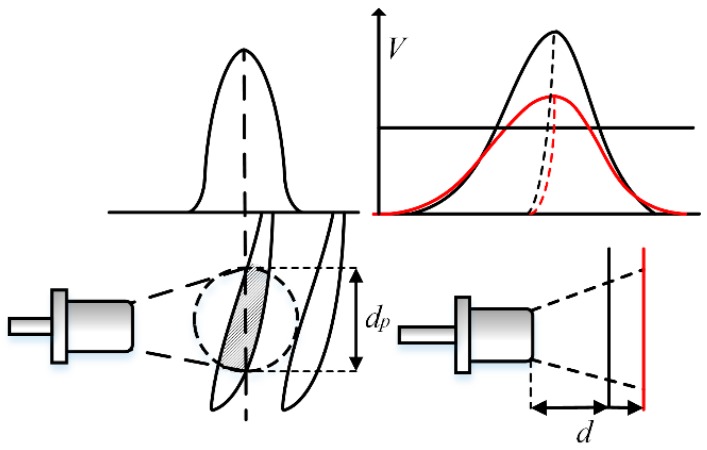
Sketch of received tip signal under different tip clearances.

Blade tip clearance varies during operation and affects the timing results. Because the microwave beam width changes with tip clearance, as Figure 6 shows, both the amplitude and the rise time of the tip signal change with tip clearance.

The peak of the sensor signal corresponds to the blade tip position with the largest RCS, and it is usually invariable with tip clearance change. Thus, the zero-crossing timing method is accurate, even when the tip clearance changes. However, this method requires that the signal has a single peak which rises relatively quickly. Some modern blades have cavities on the blade tip, and for some blades the signal peak is flat or has fluctuations. The zero-crossing timing method is not suitable for such applications.

Thus, an improved edge timing method is adopted in the proposed system. An AGC circuit is used in the signal processing circuit to stabilize the sensor signal before the comparator, thereby reducing the timing error caused by tip clearance change. The AGC circuit includes a peak detector and variable gain amplifiers, and is controlled by the microprogrammed control unit. Because the signal width is changed with tip clearance, BTT errors introduced by tip clearance variation cannot be eliminated simply by levelling the signal amplitude.

Hence, the proposed method compares both edges of the sensor signal with a fixed trigger voltage, and takes the average time of the two edges as the TOA. The impact of the clearance change on both edges is similar, so the TOA change caused by the rise time change is reduced by using a mean value of the TOA obtained from both edges. This method is equivalent to using the median of the waveform instead of one edge to trigger the timing pulse, as Figure 6 shows. The difference between the two medians is very small after amplification to the same level. In addition, the median is close to vertical when the difference between the rise and fall time is small, which makes the TOA insensitive to the amplitude change.

Another method is to sample the signal with high-speed analog-to-digital converters and process it in the host computer. The signal waveform in a certain range of amplitude is fitted and the timing sequence is calculated with different methods, such as comparing with the trigger level or finding the peak. However, this requires a high sampling rate and large transfer bandwidth, especially for high rotational speeds. The proposed system mainly uses hardware to process the signals, thereby improving the integration and reducing the resource consumption of the host computer, which is beneficial for making it an integral part of the engine health monitoring system.

## 3. Experiments

To assess the accuracy of the microwave BTT measurement system, it was compared with an existing BTT system based on a fiber optic sensor. The fiber optic BTT system used has good timing resolution and has been compared with strain gauge methods in previous experiments. For the rotor, the finite element model of which is known, the relative error of the dynamic strain is less than 10% and the relative error of the frequency is less than 1 Hz, so it can be used as a proper reference.

**Figure 7:**
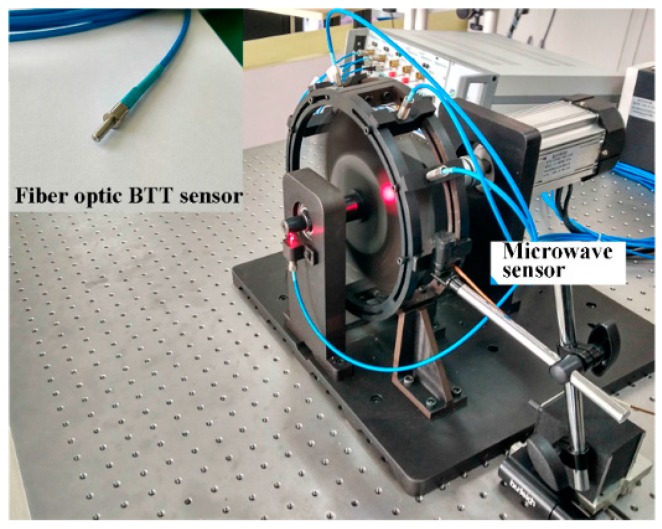
Test rig and fiber optic blade tip timing sensor (inset).

As shown in Figure 7, the experiment was conducted on a rotor test rig. The radius of the rotor was 95 mm, with 16 straight blades. The thickness of the blades was 2 mm. A low rotational speed of 1000 rpm was chosen to avoid interference from blade vibration. The effect of bending and torsion was negligible because the shaft was very short. A once-per-revolution sensor with angle divisions was installed near the bearing to minimize the errors in rotational speed. The clearance between the blade tip and microwave sensor was adjusted using a displacement platform, and the radial position of the fiber optic sensor was adjusted by shims in the fixture.

**Figure 8:**
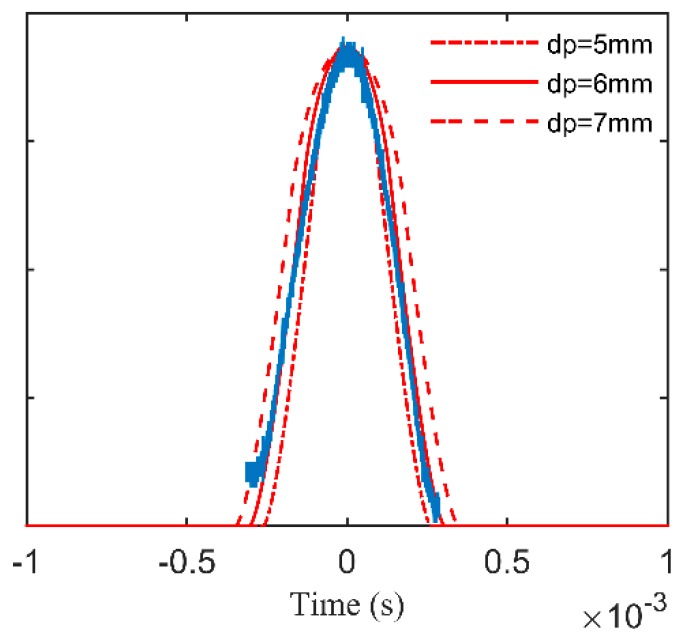
The simulation curves and the measured signal.

The blade tip of the straight blade used in the experiment was a flat plane, so the projected area of the microwave beam could be used to approximate the RCS of the blade tip. Simulation was conducted using the area of the projected region on the blade tip as the RCS. The rotational speed and the blade dimension was the same as the experiment. Figure 8 shows the simulation curves by Equation (6) and the measured signal, and the simulation result is similar to the measured signal waveform. The amplitude of the simulation curves is leveled to the measured signal waveform. The diameter of the projected region of the microwave beam *d_p_* was changed from 5 mm to 7 mm in the simulation. The result shows that the width of the signal edge was mainly affected by the projected area of the microwave beam, which was determined by the size and field distribution of the probe antenna and the tip clearance.

The pulse signal after processing by the microwave sensor was collected the same way as the optical sensors, and the rising and falling edges of the signal were acquired. The angle between the microwave sensor and the fiber optic sensor was 25°. Blades were numbered from 1 to 16.

**Figure 9:**
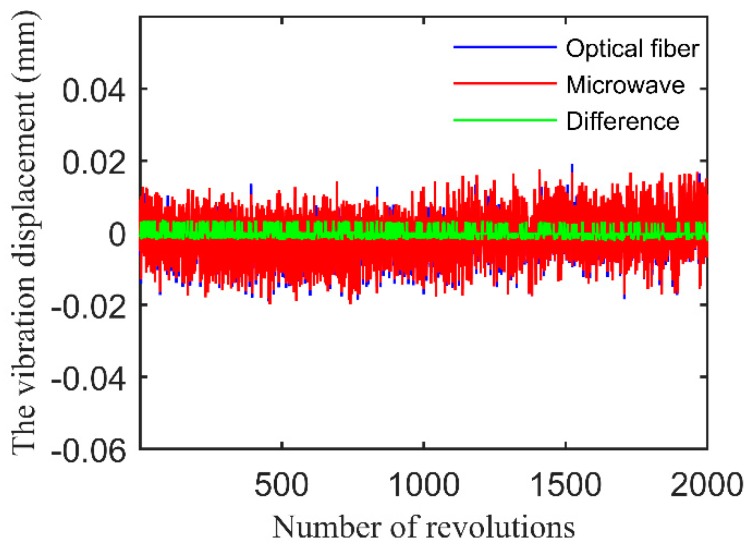
Vibration displacements of blade 1 obtained by the microwave sensor and the fiber optic sensor, and the differences between them, measured over 2000 revolutions.

The TOAs of the blades obtained by the two methods were recorded and compared. The clearance between the blade and each sensor was set to 2 mm; there was an additional 2 mm between the microwave sensor and the end of the fixture. Figure 9 shows the vibration displacements of blade 1 obtained by the microwave sensor and the fiber optic sensor, and the differences between them, measured over 2000 revolutions. The vibration displacement is the product of the TOAs and the linear velocity. The standard deviation of the vibration displacements obtained by the fiber optic sensor is 0.00203 mm, and that of the microwave sensor is 0.00226 mm. The standard deviation shows that the two BTT systems have similar random measurement errors.

Usually the tip clearance of an aero engine is within 3 mm, and it is preferred that the clearance between the sensor and the blade be larger to ensure that the blade will not hit the sensor accidentally, so the tip clearance of the microwave sensor was set to 2, 3 and 4 mm; the position of the fiber optic sensor remained unchanged. The vibration displacements of blade 1 were recorded; the means and standard deviations of the differences between the vibration displacements obtained by the two systems are shown in Table 1.

The results show that the mean of differences between the vibration displacements of the fiber optic sensor and those of the microwave sensor remain about the same when the tip clearance changes. The standard deviation is small and changes little with tip clearance. The increase of the standard deviation is mainly due to the decline of signal-to-noise ratio (SNR) with the received power. Thus, the tip clearance change has little effect on the accuracy of the microwave BTT system. The rise time of the fiber optic sensor signal is less than that of the microwave sensor, which indicates a better spatial resolution. However, the accuracy of the microwave sensor is not significantly lower due to the timing method described in chapter one.

**Figure 10:**
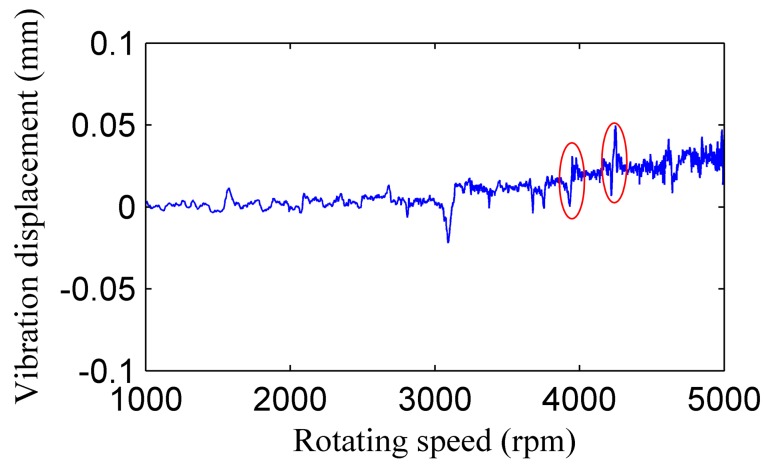
Vibration displacement of blade 1.

Figure 10 shows the vibration displacement of blade 1 measured by the microwave BTT system, when the rotating speed is raised from 1000 to 5000 rpm. The resonance regions can be found in the picture, and the blade parameters such as natural frequency can be calculated by the resonance points. For example, the engine orders of the two resonance points marked in the figure are identified by a single parameter method. The rotating speed of the resonance point at the first mark is 66.7 Hz and the order is 14, the rotating speed of the second mark is 71.9 Hz and the order is 13. The resonance frequency of the first-order bending vibration is 935.66 Hz according to the FEM model of the rotor, and the difference between the simulation result and results of the BTT system are small.

The results show that the proposed microwave sensor BTT method has good precision, and the effect of tip clearance change on the BTT accuracy is small. Thus, the microwave BTT system is a feasible and effective solution for monitoring blade vibrations in turbine engines. However, the spatial resolution of the optical sensor is better than the microwave sensor due to the small beam width, and the temperature stability of the microwave probe may also affect the measurement accuracy.

## 4. Conclusions

A BTT system based on a microwave sensor is proposed. The system uses a 24-GHz microwave sensor with an outer diameter of 8 mm and a system signal bandwidth of 10 MHz. The proposed microwave BTT system is compared with a conventional BTT system based on a fiber optic sensor. The results show that the microwave BTT system can achieve good accuracy. Thus, the system is feasible for use in applications with a polluted medium and high temperatures, where other kinds of sensor are inappropriate. The improved timing method reduced the effect of tip clearance on BTT measurement. The system has enough bandwidth for multiple blades and high rotation speeds. The proposed BTT system and probe can also be used to measure tip clearance.

## Figures and Tables

**Table 1:** Differences between the vibration displacements obtained by the two systems under different tip clearances.

Tip Clearance (mm)	Mean of Difference (mm)	Standard Deviation of Difference (mm)
2	2.711 × 10^−4^	0.00127
3	3.159 × 10^−4^	0.00149
4	3.826 × 10^−4^	0.00191
